# Sequencing *ASMT* Identifies Rare Mutations in Chinese Han Patients with Autism

**DOI:** 10.1371/journal.pone.0053727

**Published:** 2013-01-17

**Authors:** Lifang Wang, Jun Li, Yanyan Ruan, Tianlan Lu, Chenxing Liu, Meixiang Jia, Weihua Yue, Jing Liu, Thomas Bourgeron, Dai Zhang

**Affiliations:** 1 Key Laboratory of Mental Health, Ministry of Health (Peking University), Beijing, People's Republic of China; 2 Institute of Mental Health, Peking University, Beijing, People's Republic of China; 3 Peking-Tsinghua Center for Life Sciences, Beijing, People's Republic of China; 4 Human Genetics and Cognitive Functions, Institute Pasteur, Paris, France; 5 CNRS URA 2182 ‘Genes, synapses and cognition’, Institut Pasteur, Paris, France; 6 University Paris Diderot, Sorbonne Paris Cité, Human Genetics and Cognitive Functions, Paris, France; Emory University School Of Medicine, United States of America

## Abstract

Melatonin is involved in the regulation of circadian and seasonal rhythms and immune function. Prior research reported low melatonin levels in autism spectrum disorders (ASD). *ASMT* located in pseudo-autosomal region 1 encodes the last enzyme of the melatonin biosynthesis pathway. A previous study reported an association between ASD and single nucleotide polymorphisms (SNPs) rs4446909 and rs5989681 located in the promoter of *ASMT*. Furthermore, rare deleterious mutations were identified in a subset of patients. To investigate the association between *ASMT* and autism, we sequenced all *ASMT* exons and its neighboring region in 398 Chinese Han individuals with autism and 437 healthy controls. Although our study did not detect significant differences of genotypic distribution and allele frequencies of the common SNPs in *ASMT* between patients with autism and healthy controls, we identified new rare coding mutations of *ASMT*. Among these rare variants, 4 were exclusively detected in patients with autism including a stop mutation (p.R115W, p.V166I, p.V179G, and p.W257X). These four coding variants were observed in 6 of 398 (1.51%) patients with autism and none in 437 controls (Chi-Square test, Continuity Correction p = 0.032, two-sided). Functional prediction of impact of amino acid showed that p.R115W might affect protein function. These results indicate that ASMT might be a susceptibility gene for autism. Further studies in larger samples are needed to better understand the degree of variation in this gene as well as to understand the biochemical and clinical impacts of ASMT/melatonin deficiency.

## Introduction

Autism spectrum disorders (ASD) are neurodevelopmental disorders characterized by deficit in social interaction, communication, and the presence of repetitive or stereotypic behaviors. These symptoms become apparent in the first three years of life. Twin studies have shown that genetic factors were a potential cause of autism [1]–[3]. The concordance rate for monozygotic twins is higher than that of dizygotic twins (60%–90% vs. 0%–10%). The estimated heritability of autism is more than 90% [3]. However, most common variants identified confer relatively small increments in risk, and explain only a small proportion of familial clustering [4]. A significant proportion of this “missing heritability” will be attributable to low-frequency variants with intermediate penetrance effects [5]. Since the ability to study individual's DNA at greater resolution improved, researchers are uncovering more rare copy number variants and other genetic events associated with autism. It will help to uncover the genetic roots of more autism cases. The causative genes are highly heterogeneous and the mutations include *de novo* mutations with high penetrance, recessive mutations and the combination of *de novo* and inherited mutations [6]. Previous researches had found rare variants in *SHANK2*, *SHANK3*, *NLGN3*, *NLGN4* and other genes in a subset of patients with ASD [7]–[9].

Three independent groups reported that a majority of patients with ASD displayed significantly lower mean concentrations of melatonin compared with age matched controls [10]–[12]. These results suggested the existence of a pineal endocrine hypofunction in autistic children. Melatonin is produced in the dark by the pineal gland. It is involved in the regulation of circadian and seasonal rhythms, immunomodulation [13], and immune defense [14]. Psychiatric disorders such as major depressive disorder, bipolar disorder, and schizophrenia are associated with sleep disorders, and sleep disturbance is one of the prodromal symptoms in the recurrence of these disorders [15].

Two enzymes convert serotonin into melatonin. One is the AA-NAT (arylalkylamine N-acetyltransferase). The other is ASMT (acetylserotonin methyltransferase) which is involved in the last step of melatonin synthesis [16]. *ASMT* is located in the pseudoautosomal region 1 (PAR1) on the tip of the short arms of the X and Y chromosomes. A previous mutation screening of *ASMT* in 250 individuals affected with ASD and 255 controls identified non-conservative variations of *ASMT* including a splice site mutation (IVS5+2T<C) disrupting the ASMT activity [17]. The allele frequencies of two single nucleotide polymorphisms (SNP) rs4446909 and rs5989681 located in the promoter of *ASMT* in ASD were significantly different from those in controls (p = 0.0006, and p = 0.007, respectively). A risk haplotype GGGC in the promoter (rs4446909, rs5989681, rs56690322, and rs6644635) was more frequent in patients with ASD (p = 0.002; Odd Ratio = 1.3) and more transmitted by parents to their affected children (dominant model, p = 0.02) [17]. Moreover, three studies have indicated that the *ASMT* transcript level was found to be associated with these SNPs (rs4446909 and rs5989681) [17]–[19]. One independent study on diverse European populations could not find significant genetic association despite the observation of 3 splice site mutations (IVS5+2T<C) and one stop mutation in 390 patients with ASD compared with 1 mutation (IVS5+2T<C) in 490 controls [20]. Another research group reported a partial duplication of *ASMT* in 6–7% of cases and only 2% of controls [21]. Recently, mutation screening of *ASMT* in patients with intellectual disability (ID) was performed [22]. The results does not support *ASMT* as a causative gene for ID, though ASMT activity in B lymphoblastoid cell lines from patients carrying the mutations was significantly lower compared with controls.

To investigate whether *ASMT* was associated with autism, we performed a case-control study in Chinese Han population.

## Materials and Methods

### Ethics Statement

This study was approved by the Ethics Committee of the Institute of Mental Health, Peking University. All subjects provided written informed consent for participation in this study. Written informed consents for children were obtained from their legal guardians.

### Subjects

The sample for this study consisted of 398 children affected with autism and 437 healthy controls. These probands and controls were recruited at the Institute of Mental Health, Peking University, China. Among the 398 patients with autism, 367 were male and 31 were female. The age of the children at the time of testing ranged from 2 years to 17 years. The assessments of autism were established by two senior psychiatrists using DSM-IV criteria, Autism Behavior Checklist (ABC), and Childhood Autism Rating Scale (CARS). Exclude criteria included children with fragile X syndrome, tuberous sclerosis, a previously identified chromosomal abnormality by karyotyping analysis, and non-Han Chinese ancestry. Controls were eligible for inclusion if they denied any history of psychiatric disorders. The control samples used in this study consisted of 437 (406 males and 31 females) Chinese Han subjects. Most controls and case were born in the North of China.

Blood was obtained from children with autism and healthy controls.

### Genotyping

Genomic DNA was extracted using QIAamp DNA Blood Mini Kit (Qiagen). Information on SNPs was obtained from the dbSNP (http://www.ncbi.nlm.nih.gov/SNP/) and the international HapMap project (http://www.hapmap.org/). Considering the previously reported positive findings, we sequenced the promoter and all exons of *ASMT*. For the promoter and exon 1, the primer sequences were 5′- GCTGGCATCTTGATGTTGAA -3′ (sense) and 5′- CAACAATGGAACGTGAGTGTG-3′ (antisense). To explore whether autistic children carried the splice site mutation (IVS5+2T>C), we sequenced the exon 5 and its neighboring region. Primer sequences were 5′-TCCGTTCTCAACAGGGGGT-3′ (sense) and 5′-TGCTCGCAGAGGAGATGTTTG-3′ (antisense). This PCR fragment includes 6 SNPs (rs144935309, rs147969184, rs141937160, rs145804175, rs145494220, and rs28675287) and one splice site mutation IVS5+2T>C. The other exons (exon 2, exon 3, exon 4, exon 6, exon 7, exon 8, and exon 9) were sequenced too. The information of primers for all exons in *ASMT* and annealing temperature was listed in Table S1. The PCR amplification was performed in a 25 µl volume containing 10 mM Tris-HCl (pH 8.3), 50 mM KCl, 1.5 mM MgCl_2_, 200 µM of each dNTP, 0.3 µM of each primer, 0.6 U of Hotstart *Taq* DNA polymerase, and 30 ng of the genomic DNA. The conditions used for PCR amplification were an initial denaturation phase at 94°C for 5 min, followed by 36 cycles at 94°C for 30 sec, annealing at 55°C–61°C for 30 sec, and extension at 72°C for 40 sec, followed by a final extension phase at 72°C for 10 min.

PCR products were sequenced respectively by DNA sequencing after cleaning the PCR products using a BigDye Terminator Cycle Sequencing Ready Reaction Kit with Ampli *Taq* DNA polymerase (PE Biosystem). The fragments were separated by electrophoresis on an ABI PRISM genetic analyzer (Applied Biosystem, Foster City. U.S.A.).

### DNA analysis and prediction the effects of coding non-synonymous variants on protein function

Sequences were analyzed using the Megalign program with the Lasergene sequence analysis software package (DNAStar, Madison, WI, USA). The possible functional impact of amino acid changes was predicted by the PolyPhen-2 (Polymorphism Phenotyping) [23] (http://genetics.bwh.harvard.edu/pph2/) which is an automatic tool for prediction of possible impact of an amino acid substitution on the structure and function of a human protein. SIFT (sorts intolerant from tolerant) (http://sift.bii.a-star.edu.sg/www/SIFT_BLink_submit.html) was also used for predict possible functional impact of amino acid changes based on sequence homology [24], [25]. To investigate whether the identified variants were present in natural or autism-free populations in other database, we explore the Exome Variant Server from the National Heart, Lung, and Blood Institute (NHLBI) 1000 genomes project (http://evs.gs.washington.edu/EVS/) [26]. Identification of possible transcription factors binding sites was performed using Match-1.0 public (http://www.gene-regulation.com/pub/programs.html), TFsitescan (http://www.ifti.org/cgi-bin/ifti/Tfsitescan.pl), and TFSEARCH (Searching Transcription Factor Binding Sites). These methods all used a library from TRANSFAC® Public 6.0 [27], [28].

### Statistical Analysis

Deviation from Hardy-Weinberg Equilibrium (HWE) for genotype frequency distributions was tested using the chi-square goodness-of-fit test. All those with frequencies of minor alleles greater than 5% were used as genetic markers in this study.

The Haploview program (version 4.0) was used to determine pairwise *D*′ linkage disequilibrium (LD) to detect haplotype block analysis using the option of determining blocks based on the criteria defined by Gabriel et al [29], [30]. Allele and genotype frequencies for each polymorphism were compared between patients and controls using Pearson's chi-square analysis. The Fisher's exact test was used instead of the Pearson's chi-square test when calculated minimum expected count was less than 1. In addition, continuity correction of chi-square was used when calculated minimum expected count was less than 5 but more than 1. The SPSS statistical software was used for all analyses, and significance was set at p<0.05 (2-sided).

The power of sample size for association tests was evaluated using the Genetic Power Calculator program (http://pngu.mgh.harvard.edu/~purcell/gpc/). For the disease locus, a prevalence of 0.006, a genotype relative risk Aa = 1.5 and AA = 1.5, and a D-prime = 1 were used to perform the analyses.

## Results

### Quality control

The genotype distributions of the detected common variants (minor allele frequency more than 0.05) did not deviate from Hardy-Weinberg equilibrium (p>0.05) except SNP rs6588809. The power of common SNPs in our study was between 0.51 and 0.76. All detected rare variants were validated by resequencing both strands.

### Common variants association and haplotype analysis

We sequenced the promoter and all exons of *ASMT* in 398 children with autism and 437 healthy controls. The promoter and exon1 region includes three relatively frequent SNPs (rs4446909, rs5989681, and rs6644635). Other common SNPs with minor allele frequency (MAF) more than 0.05 were rs28675287 (intron 5), rs6588809 (exon 6), rs28613362 (intron 6), and rs11346829 (intron 8). We did not detect significant differences of genotypic distribution and allele frequencies of these SNPs of *ASMT* between patients with autism and healthy controls (Table 1).

**Table 1:** Genotype and allele frequencies of common variants detected in *ASMT* between patients with autism and healthy controls.

Marker	Genotype and frequency			χ^2^ (df = 2)	*P*	Allele and frequency		χ^2^ (df = 1)	*P*	OR (95%CI)
**Promoter and exon 1**										
rs4446909	GG	AG	AA			G	A			
patients	199 (0.500)	161 (0.405)	38 (0.095)	3.015	0.221	559 (0.702)	237 (0.298)	0.950	0.330	1.109
controls	194 (0.448)	201 (0.464)	38 (0.088)			589 (0.680)	277 (0.320)			(0.900–1.366)
rs5989681	GG	CG	CC			G	C			
patients	128 (0.326)	193 (0.491)	72 (0.183)	2.687	0.261	449 (0.571)	337 (0.429)	1.729	0.188	1.139
controls	119 (0.274)	231 (0.531)	85 (0.195)			469 (0.539)	401 (0.461)			(0.938–1.383)
rs6644635	CC	CT	TT			C	T			
patients	232 (0.584)	147 (0.370)	18 (0.046)	0.485	0.785	611 (0.770)	183 (0.230)	0.020	0.887	1.017
controls	257 (0.588)	156 (0.357)	24 (0.055)			670 (0.767)	204 (0.233)			(0.810–1.277)
**Intron 5**										
rs28675287	TT	CT	CC			T	C			
patients	147 (0.375)	187 (0.477)	58 (0.148)	1.719	0.423	481 (0.614)	303 (0.386)	0.752	0.386	1.091
controls	144 (0.333)	224 (0.519)	64 (0.148)			512 (0.593)	352 (0.407)			(0.896–1.330)
**Exon 6**										
rs6588809^a^	TT	CT	CC			T	C			
patients	105 (0.276)	185 (0.487)	90 (0.237)	4.052	0.132	395 (0.520)	365 (0.480)	0.692	0.405	0.920
controls	91 (0.222)	226 (0.553)	92 (0.225)			408 (0.499)	410 (0.501)			(0.755–1.120)
**Intron 6**										
rs28613362	AA	AG	GG			A	G			
patients	225 (0.581)	137 (0.354)	25 (0.065)	0.961	0.618	587 (0.758)	187 (0.242)	0.698	0.403	0.906
controls	255 (0.601)	148 (0.349)	21 (0.050)			658 (0.776)	190 (0.224)			(0.720–1.141)
**Intron 8**										
rs11346829	GG	G/del	del/del			G	del			
patients	346 (0.880)	47 (0.120)	0	3.167	0.273^b^	739 (0.940)	47 (0.060)	0.002	0.966	0.991
controls	382 (0.888)	45 (0.105)	3 (0.007)			809 (0.941)	51 (0.059)			(0.659–1.492)

a , Hardy-weinberg Equilibrium *P* value less than 0.05 in healthy controls;

b , Monte Carlo significance (2-sided). OR, odds ratio; CI, confidence interval; del, deletion.

**Figure 1:**
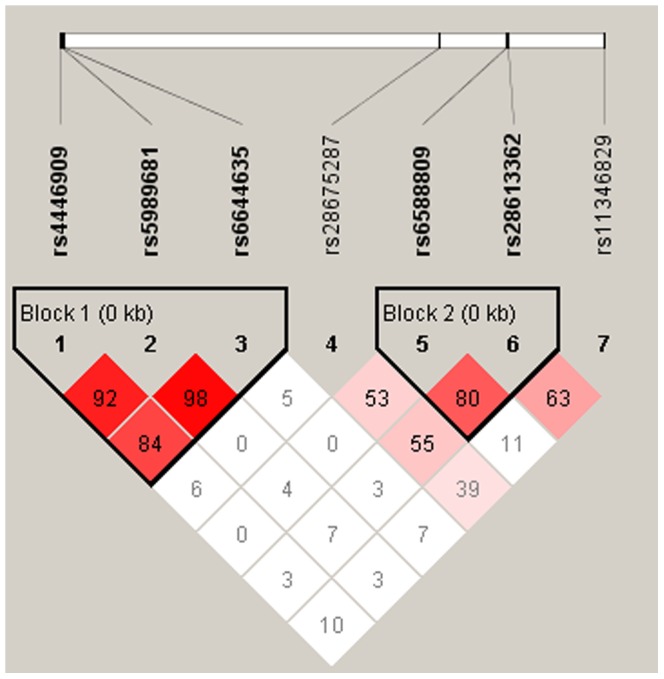
Linkage disequilibrium (LD) block of 4 common SNPs in promoter of *ASMT*. Markers with Linkage disequilibrium (LD) (*D*′<1 and LOD>2) are shown in red through pink (color intensity decreases with decreasing *D*′ value). *D*′ value shown within each square represents a pairwise LD relationship between the two polymorphisms. This LD plot was generated with the Haploview.

In our study, pair-wise linkage disequilibrium (LD) analysis was performed for the common SNPs (rs4446909, rs5989681, rs6644635, rs28675287, rs6588809, rs28613362, and rs11346829). Two blocks were identified. Three SNPs (rs4446909, rs5989681, and rs6644635) were in one block with *D*′ ranged from 0.84 to 0.98 (Figure 1). The other block was constructed by rs6588809 and rs28613362 (*D*′ = 0.80). Therefore we performed haplotype analysis for these two blocks. No significant difference of haplotype frequencies between cases and controls was found. These results were listed in Table 2. To homogenize and compare the results with previous study, we also performed haplotype analysis constructed by 4 SNPs in promoter (rs4446909, rs5989681, rs56690322, and rs6644635). The frequency of the risk haplotype GGGC was 0.34 in patients and 0.30 in controls (p = 0.081; OR = 1.203 (95%CI: 0.977–1.481)) (Table S2).

**Table 2:** Comparison of haplotype frequencies between patients with autism and healthy controls.

Haplotype	Case (freq)	Control (freq)	*P*	OR (95%CI)
Haplotype 1^a^				
GGC	268.32 (0.341)	263.09 (0.304)	0.083	1.201 (0.976–1.478)
ACC	220.51 (0.281)	267.72 (0.310)	0.235	0.879 (0.711–1.087)
GGT	169.12 (0.215)	194.64 (0.225)	0.690	0.954 (0.755–1.205)
GCC	113.55 (0.144)	130.28 (0.151)	0.775	0.961 (0.731–1.262)
Haplotype 2^b^				
CA	344.62 (0.456)	390.39 (0.478)	0.394	0.916 (0.750–1.120)
TA	228.38 (0.302)	241.61 (0.296)	0.766	1.034 (0.832–1.284)
TG	164.62 (0.218)	166.39 (0.204)	0.482	1.091 (0.856–1.392)

a , Haplotype constructed by rs4446909, rs5989681, and rs6644635;

b , Haplotype constructed by rs6588809 and rs28613362; Freq, frequency; OR, odds ratio; CI, confidence interval.

### Rare nonsynonymous coding variants detected and prediction of functional effects

No genetic variants were detected in exon 2, exon 4 and exon 9. However, a few coding variants were indentified in exon1, exon3, exon5, exon7, and exon8. All coding variants identified in present research were listed in Table 3.

**Table 3:** Rare coding variants identified in *ASMT*.

Individuals	Mutation	Genotype	Cases	Controls	Inheritance	Prediction of possible functional impact		Exome Variant Server^e^	
			(n = 398)	(n = 437)	status^a^	Polyphen 2 HumDiv^b^	SIFT^c^	Allele Count^f^	MAF^g^ (EA/AA/All)
Autism only									
	p.R115W	CT	2	0	Mother	Possibly damaging	Affect protein function	-	-
	p.V166I	AG	1	0	Mother	Benign	Tolerated	EA:A = 2/G = 8590AA:A = 0/G = 4406All: A = 2/G = 12996	0.0233/0/0.0154
	p.V179G	GT	2	0	MotherFather	Benign	Tolerated	-	-
	p.W257X	AG	1	0	Father	Damaging	Neutral^d^	-	-
Autism and controls									
	rs17149149 (p.N17K)	AC	18	30	N.A	Possibly damaging	Tolerated	EA:A = 2/G = 8588AA:A = 9/G = 4393All:A = 11/G = 12981	0.0233/0.2045/0.0847
	p. G151S	AG	7	9	N.A	Benign	Tolerated	EA:A = 2/G = 8590AA:A = 1/G = 4405All:A = 3/G = 12995	0.0233/0.0277/0.0231
	p.I211M	CG	1	1	Mother	Possibly damaging	Affect protein function	-	-
	p.P243L	CT	1	1	Father	Probably damaging	Affect protein function	EA:T = 3/C = 8589AA:T = 2/C = 4404All:T = 5/C = 12993	0.0349/0.0454/0.0385
Controls only									
	rs17149149 (p.N17K)	AA	0	1	N.A	Possibly damaging	Tolerated	EA:A = 2/G = 8588AA:A = 9/G = 4393All:A = 11/G = 12981	0.0233/0.2045/0.0847
	p.T217M	CT	0	1	N.A	Possibly damaging	Tolerated	EA :T = 3/C = 8589AA :T = 2/C = 4404All :T = 5/C = 12993	0.0349/0.0454/0.0385

a , Inheritance status only for cases;

b , The possible functional impact of amino acid changes was predicted by the PolyPhen 2 program HumDiv model;

c , Functional prediction performed by SIFT;

d , Functional prediction performed by SIFT indel;

e , Mutations found in database of Exome Variant Server;

f , The observed allele counts for the listed alleles (delimited by/);

g , The minor-allele frequency in percent listed in the order of European American (EA), African American(AA) and all populations (All) (delimited by/). N.A, data was not available.

A relatively frequent non-synonymous variant rs17149149 (p.N17K) was observed in 18 patients (all heterozygotes) and in 31 controls (30 heterozygotes and one homozygote). However, the frequencies of allele and genotype were not significantly different between patients and health controls.

**Figure 2:**
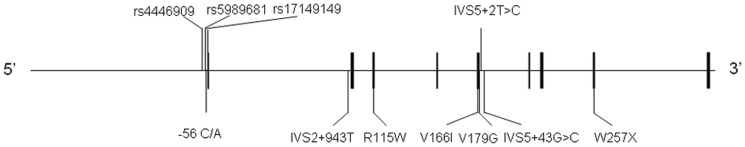
Localization of rare nonsynonymous variants or splite site identified in patients with autism. Upper: SNPs were associated with autism and rare variants identified in children with autism in other researches. Lower: rare nonsynonymous only identified in children with autism not in controls in our research.

Among the 22 rare variants identified, 9 were affecting the ASMT protein sequence: 8 were nonsynonymous (p.N17K, p.R115W, p.G151S, p.V166I, p.V179G, p.I211M, p.T217M, and p.P243L) and 1 was a stop mutation (p.W257X) (Table 3). The locations of rare nonsynonymous variants identified in patients with autism were shown in Figure 2 and Figure S1, S2, S3, S4, S5, S6. Four nonsynonymous variants (p.R115W, p.V166I, p.V179G, and p.W257X) were only found in children with autism. These coding rare variants in autism were inherited. Three variants (p.R115W, p.V179G, and p.W257X) were not present in database such as Exome Variant Server. Moreover, functional prediction of impact of amino acid was performed using two pieces of software. The consistent results showed that p.R115W might affect protein function.

Importantly, the four variants (p.R115W, p.V166I, p.V179G, and p.W257X) affecting the protein sequence were observed in 6 of 398 (1.51%) patients with autism and none in 437 controls (Chi-Square test, Continuity Correction p = 0.032, two-sided).

Four rare variants were observed in patients and controls (rs17149149, p.G151S, p.I211M and p.P243L), and 1 was observed only in controls (p.T217M).

### Rare noncoding variants and synonymous variants identified

In promoter and exon 1, we detected six rare noncoding variants such as rs56690322 (P1BC), −91G/A, −56C/A, −45C/T, +11C/G, and +57G/C with MAF less than 0.05. They are located 91 bp (−91, biallele G/A), 56 bp (−56, biallele C/A), 45 bp (−45, biallele C/T) upstream from the transcription site (+1), and 11 bp (+11, biallele C/G) and 57 bp (+57, biallele G/C) downstream from the transcription site respectively (Figure S1). Among these rare noncoding variants, one variant (−56C/A) was exclusively detected in one patient with autism (Table 4). However, it did not alter the binding of transcription factors.

**Table 4:** Rare noncoding and synonymous variants identified in ASMT.

Location	Mutation	Genotype	Cases (n = 398)	Controls (n = 437)
Promoter	rs56690322	AG	6	4
	−91G/A	AG	0	1
	−56C/A^a^	CA	**1**	0
	−45C/T^a^	CT	20	26
		TT	1	2
Exon 1	+57G/C	GC	0	1
	+11 C/G^a^	CG	21	28
		GG	0	2
	+147 A/C	AC	9	6
Intron 2	IVS2+943T	TT	**1**	0
Exon 3	p.S91S	CT	0	1
	H119H	CT	0	1
Intron 5	IVS5+28G>A	AG	4	2
	IVS5+43G>C	CG	**1**	0
Intron 7	IVS7+22 A>T	AT	1	1
Exon 8	F237F	CT	0	1

a , variants detected in previous research [17].

Other nonconding and synonymous variants were located in exons or at the vicinity of the exons (Table 4). The localization of splite site identified in patients with autism was shown in Figure 2. Among these variants, two variations including IVS2+943T (insertion, intron 2) and IVS5+43G>C (intron 5) were detected only in patients with autism. However, the frequencies of allele and genotype were not significantly different between patients and health controls (Table S3). All rare variants were shown in additional Figure S1, S2, S3, S4, S5, S6.

One healthy control, who was homozygous for the genotype AA for rs17149149 (p.N17K), had combination of variants −45C/T (TT) and +11C/G (GG). Another healthy control that carries the genotype AC for rs17149149 had the same combination of variants −45C/T (TT) and +11C/G (GG). We also detected one patient who had the combination of variants of TT for −45 C/T, CG for +11 C/G variant, and AC for rs17149149 (Table S4).

## Discussion

Melatonin plays roles in neurogenesis [31], immune defense [14], circadian rhythms and sleep [16]. Moreover, brain melatonin production may have a crucial role in fetal stages of development as the brain tissue is highly sensitive to free radial damage because of its high utilization of oxygen [32], [33]. Previous studies reported that a subset of patients with psychiatric disorders display significant difference in the onset, offset, and duration of melatonin secretion and production compared with the healthy controls [34]–[36]. Furthermore, recent studies detected that mutations in *ASMT* were associated with patients with attention-deficit/hyperactivity disorder (ADHD), and schizophrenia [37], [38].

A subset of patients with ASD had low melatonin levels suggesting pineal hypofunction [11]. Therefore, it was hypothesized that abnormal melatonin levels might play a role in the brain development and increase the risk of affecting autism. The genetic variability of *ASMT* was investigated. The polymorphisms (rs4446909 and rs5989681) located in promoter of *ASMT* were associated with ASD. Following this study, the same risk alleles were associated with depression [19], [39] and bipolar disorders [18]. Moreover, these two SNPs were associated with a decrease in *ASMT* transcripts in blood cell lines [17]–[19]. However, we did not detect the significant association between these two and other common SNPs and autism. The MAF of rs56690322 was only 0.008 in the Chinese Han population compared with 0.1 in European populations.

Melke and colleagues observed the p.N17K mutation in one individual with ASD. This mutation was present in the SNP database at a frequency of 0.4–0.7% in Han Chinese, though Han Chinese was not included in their control samples [17]. Our studies identified p.N17K variant which was observed in 18 patients (all heterozygote) and in 31 controls (30 heterozygotes and one homozygote with genotype AA). The frequencies of allele and genotype in patients were not significantly different from those in healthy controls. As the sample size of our study is relatively large, it is indicated that p.N17K mutation is not associated with autism in Chinese Han population. Biochemical analyses indicated that the p.N17K variant might disrupt the ASMT activity (<4% of the wild type ASMT activity) [40]. It is not clear whether a heterozygous deleterious mutation of ASMT can dramatically reduce the melatonin synthesis *in vivo*. However, the control homozygous for the p.N17K is most likely deficient for melatonin. Unfortunately, we have no detailed clinical information on the patients and controls that could allow us to detect intermediate phenotype associated with the presence of *ASMT* deleterious mutations.

One splice site mutation in *ASMT* (IVS5+2T>C) was reported by two independent study [41], [17]. Our study did not detect this variant in 398 cases and 437 controls in Chinese Han population. One possible reason is that the genetic heterogeneity exits between different ethnic populations. However, we detected nine rare coding variants. Four variants (p.R115W, p.V166I, p.V179G, and p.W257X) affecting the protein sequence were observed in 6 of 398 (1.51%) patients with autism and none in 437 controls (Chi-Square test, Continuity Correction p = 0.032, two-sided). Among these variants, three variants (p.R115W, p.V179G, and p.W257X) were not present in natural or autism-free populations by using database from the National Heart, Lung, and Blood Institute (NHLBI) 1000 genomes project. These variants were not reported by recent exome sequencing researches in ASD [42]–[44]. Moreover, results of functional prediction showed that p.R115W might affect protein function. Further functional experiments are needed to confirm it.

Our research has several limitations. First one is the sample size. Although our sample size is relatively large, it is necessary to expand the sample size to detect rare variants. Indeed, none of the large scale genome wide association studies reported in ASD could detect strong reproducible susceptibility genes for the disorder. The second limitation is the lack of information on the clinical and biochemical impacts of the ASMT deleterious variants and/or SNPs. It would be important in the future to ascertain whether the ASMT variants identified in this study are associated with intermediate phenotypes such as low melatonin level, ASMT deficiency and sleep problems. Finally, we did not sequence other genes in melatonin pathway, which might be involved in the normal function of melatonin.

In summary, we performed a case-control study to explore the association between *ASMT* genetic variants and autism. Our research ascertained for the first time the variability of *ASMT* in a relatively large sample of patients and controls from Han Chinese descents. We could identify new rare deleterious variants and ascertain the promoter genetic variability. Four coding variants in *ASMT* exclusively detected in a subset of patients with autism might be associated with autism risk. Further studies are necessary to understand the impact of ASMT/melatonin deficiency at the clinical level.

## Supporting Information

Table S1 **Details of PCR primers and conditions for sequencing promoter and exons of** ***ASMT*** **.**
(DOC)

Table S2 **Comparison of haplotype frequencies between patients with autism and healthy controls.**
(DOC)

Table S3 **Genotype and allele frequencies of rare noncoding variants in** ***ASMT*** **between patients with autism and healthy controls.**
(DOC)

Table S4 **Individuals carried rare variants both in the promoter and exon 1 of** ***ASMT*** **.**
(DOC)

Figure S1 **Variations detected in the promoter and exon 1 of** ***ASMT*** **.**
(DOC)

Figure S2 **Variations detected in exon 3 of** ***ASMT*** **.**
(DOC)

Figure S3 **Variations detected in exon 5 and its neighboring region of** ***ASMT*** **.**
(DOC)

Figure S4 **Variations detected in exon 6 and its neighboring region of** ***ASMT*** **.**
(DOC)

Figure S5 **Variations detected in exon 7 and its neighboring region of** ***ASMT*** **.**
(DOC)

Figure S6 **Variations detected in exon 8 and its neighboring region of** ***ASMT*** **.**
(DOC)
