# Supplementary material for: Sequencing ASMT Identifies Rare Mutations in Chinese Han Patients with Autism
Source: PLoS One. 2013 Jan 17;8(1):e53727. doi: 10.1371/journal.pone.0053727 (PMC3547942; doi:10.1371/journal.pone.0053727)
Supplement: Table S1 — Details of PCR primers and conditions for sequencing promoter and exons of ASMT . (DOC) [file pone.0053727.s001.doc]

**Table S1. Details of PCR primers and conditions for sequencing promoter and exons of *ASMT***

| Position | Primers | PCR product length (bp) | T  (°C) |
| --- | --- | --- | --- |
| Promoter & Exon 1 | Forward: 5'- GCTGGCATCTTGATGTTGAA -3'  Reverse: 5'- CAACAATGGAACGTGAGTGTG-3' | 586 | 55 |
| Exon 2 | Forward: 5'-CTTTCCTCCCTAGCCTGCC-3'  Reverse: 5'- TGGGCTGCAGAGGGTGTCTAT-3' | 326 | 55.5 |
| Exon 3 | Forward: 5'-GTCACCTTTGCGCCTTCCAC-3'  Reverse: 5'-TCACCTCCTCCACTGCCACTTC-3' | 977 | 61 |
| Exon 4 | Forward: 5'-CCTGGGCTACAGAGCTGAAA-3'  Reverse: 5'-CCTGGGTTGTGCCATTTGAGT-3' | 280 | 60 |
| Exon 5 | Forward: 5’-TCCGTTCTCAACAGGGGGT-3’  Reverse: 5’-TGCTCGCAGAGGAGATGTTTG-3’ | 394 | 55 |
| Exon 6 | Forward: 5'-AGCTTGCAGTGAGCGGAAAT-3'  Reverse: 5'-AGGGTCCAACCCAGGAACTTG-3' | 479 | 58.5 |
| Exon 7 | Forward: 5'-TGGGTTGGACCCTTCATGAG-3'  Reverse: 5'-TCCGGGAGTGAGAGGAAAAC-3' | 347 | 56 |
| Exon 8 | Forward: 5'-AGCCTGGAAGACCTGGGAAAG-3'  Reverse: 5'-ACCTGTGGGATGATTTCAGTG-3' | 339 | 59 |
| Exon 9 | Forward: 5'-CGGTGCCCTGACTGTCCTCT-3'  Reverse: 5'-CCATCAGCGTGGTCCTCAGTA-3' | 507 | 61 |
