# Supplementary material for: Sequencing ASMT Identifies Rare Mutations in Chinese Han Patients with Autism
Source: PLoS One. 2013 Jan 17;8(1):e53727. doi: 10.1371/journal.pone.0053727 (PMC3547942; doi:10.1371/journal.pone.0053727)
Supplement: Table S2 — Comparison of haplotype frequencies between patients with autism and healthy controls. (DOC) [file pone.0053727.s002.doc]

**Table S**2. Comparison of haplotype frequencies between patients with autism and healthy controls

| Haplotype ^a^ | Case (freq) | Control (freq) | *P* | OR (95%CI) |
| --- | --- | --- | --- | --- |
| GGGC | 267.33 (0.341) | 263.45 (0.305) | 0.081 | 1.203 (0.977-1.481) |
| ACGC | 219.54 (0.280) | 267.72 (0.310) | 0.241 | 0.880 (0.711-1.090) |
| GGGT | 163.12 (0.208) | 190.63 (0.221) | 0.622 | 0.942 (0.744-1.194) |
| GCGC | 113.53 (0.145) | 129.92 (0.150) | 0.830 | 0.970 (0.738-1.275) |

a, Haplotype constructed by rs4446909, rs5989681, rs56690322, and rs6644635.
