# Supplementary material for: Sequencing ASMT Identifies Rare Mutations in Chinese Han Patients with Autism
Source: PLoS One. 2013 Jan 17;8(1):e53727. doi: 10.1371/journal.pone.0053727 (PMC3547942; doi:10.1371/journal.pone.0053727)
Supplement: Table S3 — Genotype and allele frequencies of rare noncoding variants in ASMT between patients with autism and healthy controls. (DOC) [file pone.0053727.s003.doc]

Table S3. Genotype and allele frequencies of rare noncoding variants in ASMT between patients with autism and healthy controls
Marker	Genotype and frequency	¦Ö2
(df=2)	p	Allele and frequency	¦Ö2
(df=1)	P
	OR
(95%CI)	
Promoter and exon 1											
rs56690322	GG 	AG	AA			G 	A				
patients	391 (0.985)	6 (0.015)	0	0.222b	0.637c	788 (0.992)	6 (0.008)	0.221	0.638c	0.604	
controls	433 (0.991)	4 (0.009)	0			870 (0.995)	4 (0.005)			(0.170-2.148)	
-45C/Ta	CC	CT	TT			C	T				
patients	377 (0.947)	20 (0.050)	1 (0.003)	0.598	0.736d	774 (0.972)	22 (0.028)	0.617	0.432	1.251	
controls	409 (0.936)	26 (0.059)	2 (0.005)			844 (0.966)	30 (0.034)			(0.715-2.186)	
 +11C/G	CC	CG	GG			C	G				
patients	376 (0.947)	21 (0.053)	0	2.314	0.401d	773 (0.974)	21 (0.026)	1.397	0.237	1.399	
controls	407 (0.931)	28 (0.064)	2 (0.005)			842 (0.963)	32 (0.037)			(0.800-2.447)	
+147 A/C	AA	AC	CC			A	C				
patients	388 (0.977)	9 (0.023)	0	0.941b	0.332	785 (0.989)	9 (0.011)	0.933	0.334	0.603	
controls	431 (0.986)	6 (0.014)	0			868 (0.993)	6 (0.007)			(0.214-1.702)	
Intron 5											
IVS5+28G>A	GG	AG	AA			G	A				
patients	385 (0.990)	4 (0.010)	0	0.288b	0.592c	774 (0.995)	4 (0.005)	0.287	0.592c	0.450	
controls	429 (0.995)	2 (0.005)	0			860 (0.998)	2 (0.002)			(0.082-2.464)	
IVS5+43G>C	GG	CG	CC			G	C				
patients	390 (0.997)	1 (0.003)	0	1.104b	0.476e	781 (0.999)	1 (0.001)	1.103	0.476d	-	
controls	431	0	0			862	0				
Intron 6											
rs7471973	CC	CT	TT			C	T				
patients	349 (0.904)	36 (0.093)	1 (0.003)	1.196	0.710d	734 (0.951)	38 (0.049)	0.286	0.593	0.881	
controls	387 (0.913)	37 (0.087)	0			811 (0.956)	37 (0.044)			(0.554-1.401)	
OR, odds ratio; CI, confidence interval; del, deletion; a, Hardy-weinberg Equilibrium P value less than 0.05 in healthy controls; b, df=1; 
c, continuity correction of chi-square test was used; d, Monte Carlo significance (2-sided); e, Fisher's exact test was used.
