# Supplementary material for: Sequencing ASMT Identifies Rare Mutations in Chinese Han Patients with Autism
Source: PLoS One. 2013 Jan 17;8(1):e53727. doi: 10.1371/journal.pone.0053727 (PMC3547942; doi:10.1371/journal.pone.0053727)
Supplement: Table S4 — Individuals carried rare variants both in the promoter and exon 1 of ASMT . (DOC) [file pone.0053727.s004.doc]

**Table S4. Individuals carried rare variants both in the promoter and exon 1 of *ASMT***

| Individual | -45C/T | +11C/G | rs17149149 |
| --- | --- | --- | --- |
| 3A (case) | TT | CG | AC |
| C184 (control) | TT | GG | AA |
| C485 (control) | TT | GG | AC |
