# Supplementary material for: Sequencing ASMT Identifies Rare Mutations in Chinese Han Patients with Autism
Source: PLoS One. 2013 Jan 17;8(1):e53727. doi: 10.1371/journal.pone.0053727 (PMC3547942; doi:10.1371/journal.pone.0053727)
Supplement: Figure S1 — Variations detected in the promoter and exon 1 of ASMT . (DOC) [file pone.0053727.s005.doc]

**Figure S1. Variations detected in the promoter and exon 1 of *ASMT***

**Exon 1**

**
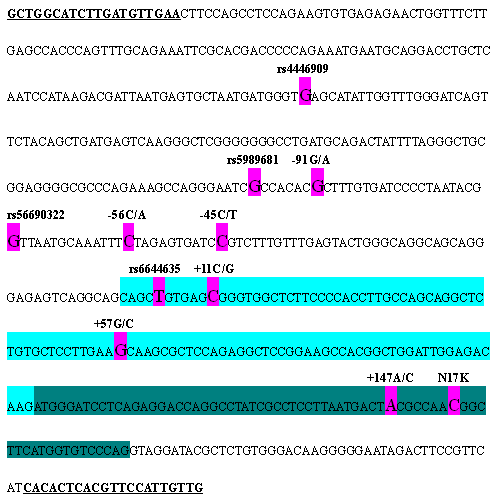
**

Primers are indicated in bold and underlined. ASMT mRNA is indicated in light and dark blue

ASMT ORF is indicated in dark blue. SNPs and rare variants are indicated in pink.
