# Supplementary material for: Sequencing ASMT Identifies Rare Mutations in Chinese Han Patients with Autism
Source: PLoS One. 2013 Jan 17;8(1):e53727. doi: 10.1371/journal.pone.0053727 (PMC3547942; doi:10.1371/journal.pone.0053727)
Supplement: Figure S3 — Variations detected in exon 5 and its neighboring region of ASMT . (DOC) [file pone.0053727.s007.doc]

**Figure S3. Variations detected in exon 5 and its neighboring region of *ASMT***


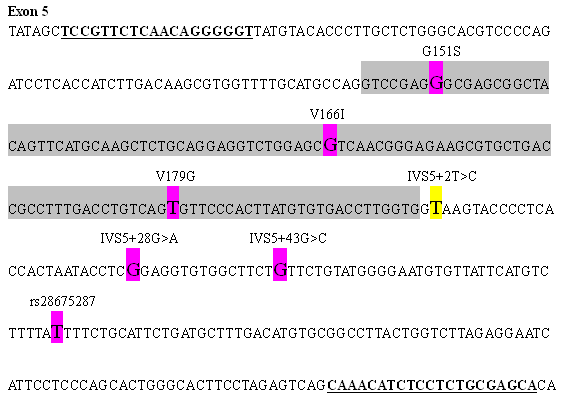


Primers are indicated in bold and underlined. Exon is indicated in gray. SNPs and rare variants are indicated in pink. Splice site which was not detected in our research is indicated in yellow.
