# Supplementary material for: Sequencing ASMT Identifies Rare Mutations in Chinese Han Patients with Autism
Source: PLoS One. 2013 Jan 17;8(1):e53727. doi: 10.1371/journal.pone.0053727 (PMC3547942; doi:10.1371/journal.pone.0053727)
Supplement: Figure S4 — Variations detected in exon 6 and its neighboring region of ASMT . (DOC) [file pone.0053727.s008.doc]

**Figure S4. Variations detected in exon 6 and its neighboring region of *ASMT***


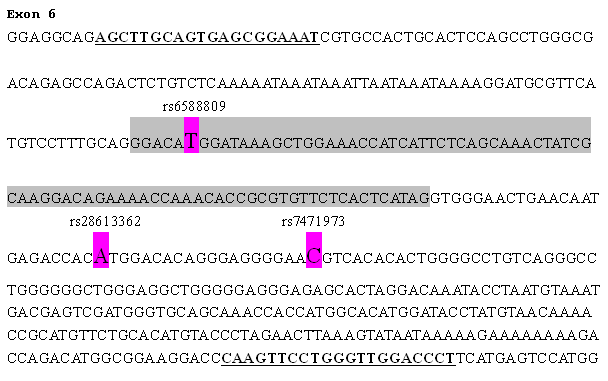


Primers are indicated in bold and underlined. Exon is indicated in gray. SNPs and rare variants are indicated in pink.
